# The effects of acute aerobic exercise on inhibitory control and resting state heart rate variability in children with ADHD

**DOI:** 10.1038/s41598-020-76859-9

**Published:** 2020-11-17

**Authors:** Chien-Lin Yu, Shu-Shih Hsieh, Ting-Yu Chueh, Chung-Ju Huang, Charles H. Hillman, Tsung-Min Hung

**Affiliations:** 1 Department of Physical Education, National Taiwan Normal University, No.162, Section1 Ho-Ping East Road, Taipei, 106 Taiwan; 2 Department of Psychology, Northeastern University, Boston, MA USA; 3 Graduate Institute of Sport Pedagogy, University of Taipei, Taipei, Taiwan; 4 Department of Physical Therapy, Movement, and Rehabilitation Sciences, Northeastern University, Boston, MA USA; 5 Institute for Research Excellence and Learning Science, National Taiwan Normal University, Taipei, Taiwan

**Keywords:** Neuroscience, Psychology, Biomarkers, Diseases

## Abstract

The current study examined the effects of acute moderate-intensity aerobic exercise (MAE) on inhibitory control and resting-state heart rate variability (HRV) in children with Attention-Deficit/Hyperactivity Disorder (ADHD). Our data show that acute MAE resulted in higher response accuracy of a modified flanker task regardless of task difficulty for 60 min (*p* = .001). Aerobic exercise further resulted in more effective conflict detection, as measured by greater amplitude (*p* = .012) and shorter latency (*p* = .029) of the N2 component of event-related brain potential, for 60 min regardless of task difficulty. In contrast, acute MAE did not modulate sympathovagal balance signified by HRV at either 30 min or 60 min following exercise cessation. Collectively, our findings suggest that the beneficial effects of acute aerobic exercise on inhibitory control are sustained for 60 min in children with ADHD. However, acute aerobic exercise may not modulate sympathovagal balance during the post-exercise recovery. Overall, we highlight the importance of acute aerobic exercise for children with ADHD as a potential means to facilitate brain health.

## Introduction

Attention-Deficit/Hyperactivity Disorder (ADHD) is considered the most common neurodevelopmental disorder among children, affecting 6% of children globally^1^. Behavioral symptoms of ADHD comprise a developmentally inappropriate pattern of inattention, impulsiveness, and/or hyperactivity^2^. At the neurocognitive level, deficits in inhibitory control as well as the underlying neural processes subserving this cognitive domain may represent the core cognitive deficits in children with ADHD^3^. Although pharmacologic treatments have proven effective in managing ADHD symptoms^4^, potential adverse effects, such as high costs^5,6^ and failure to normalize recipients over the long term^7^, argue for other complementary interventions. Accordingly, a single bout of exercise may be an efficacious and complementary intervention to improve cognitive performance in children with ADHD, as the 2018 Physical Activity Guidelines for Americans recommends the beneficial effects of acute bouts of structured exercise lasting > 20 min on childhood cognitive and brain health^8–10^.

A growing body of literature has highlighted the benefits of acute bouts of moderate-intensity aerobic exercise (MAE) on behavioral correlates of inhibitory control assessed either via flanker tasks^11,12^ or Stroop tests^13,14^. Beyond behavioral assessments of inhibitory control, it would be informative to utilize electrophysiological assessments (including event-related brain potentials [ERP]) to study the neural processes subserving inhibitory control in children with ADHD following acute MAE. ERPs afford temporally sophisticated measurement of changes in neuroelectric activity that underpin distinct cognitive operations. In particular, the N2-ERP is a component elicited between 200–400 ms following stimulus onset and maximal over the frontocentral scalp region^15^. The frontal N2 is thought to be specifically related to conflict detection when facing two competing stimuli (e.g., task-relevant vs. task-irrelevant information)^15^. Evidence has indicated that children with ADHD have smaller N2 amplitude during inhibitory control tasks when compared to their typically developing (TD) counterparts, which may imply failures in conflict detection^16,17^. Despite a paucity of research delineating the modulatory effects of acute exercise on the N2 in children with ADHD, findings from other clinical population with deficits in inhibitory control could provide hints regarding the potential effects of acute exercise. For example, studies of individuals with drug addiction (methamphetamine-dependent patients) indicates that N2 amplitude is enlarged by acute MAE^18,19^; thus, such a model may extend to children with ADHD if a similar mechanism (reflected in the N2) were to underlie attentional dysfunction across ADHD and drug addiction. Another ERP component worth investigation is the P3-ERP, a component that is elicited approximately between 300 and 700 ms following stimulus onset and is typically maximal over the centroparietal scalp region^20^. Findings suggest that acute MAE engenders increases in P3 amplitude^11,12^ during a flanker task relative to an inactive control intervention, indicating that acute MAE has benefits to attentional engagement^20^. Together, the N2 and P3 components provide insight into the temporal dynamics of inhibitory control, and how the stimulus–response process is influenced by engagement in acute MAE in children with ADHD.

To date, it remains unclear how long the transient effects of acute MAE on inhibitory control may last in children with ADHD. The majority of studies on children with ADHD examined modulations of inhibitory control either immediately after (i.e., within 5 minutes^13,14^) or after a short delay (i.e., < 15 min^11,12^) relative to exercise cessation. Such an approach, while initially compelling, precludes our understanding of the longer effects of acute MAE on inhibitory control. Moreover, there is a paucity of research addressing how acute exercise modulates subtle neural processes (e.g., N2- and P3-ERP) underlying inhibitory control across the post-exercise recovery period in children with ADHD. Therefore, more research is necessary to elucidate the sustained effects of acute MAE on inhibitory control in children with ADHD in order to gain insight into how best to implement MAE as a therapeutic intervention in managing ADHD symptoms.

In addition to behavioral and neuroelectric correlates of inhibitory control, the inclusion of measures of the autonomic nervous system (ANS), as indexed by heart rate variability (HRV), affords insight into the sustained effects of acute MAE on cognition. In the frequency domain, the low frequency component of HRV (LF-HRV) corresponds to sympathetic influence (with a parasympathetic component), whereas high frequency component of HRV (HF-HRV) corresponds to vagal-mediated respiratory sinus arrhythmia and is regarded as a marker of parasympathetic influence. The LF/HF ratio, therefore, is indicative of the balance between sympathetic and parasympathetic modulations, with decreased ratio indicative of greater parasympathetic dominance and greater ratio indicative of increased parasympathetic withdrawal or stronger sympathetic influence^21^. Given that cognitive control is regulated by brain regions (e.g., prefrontal cortex, anterior cingulate cortex, amygdala) that are also involved in the regulation of sympathetic and parasympathetic control of HR, Thayer and his colleagues proposed that higher resting HRV, as reflected by parasympathetic dominance over sympathetic influence, is associated with better prefrontal-mediated cognitions^22^. However, a growing body of literature indicated an excessive parasympathetic dominance in children with ADHD relative to their typically developing counterparts^23,24^, probably as a result of impaired functioning in the locus coeruleus-norepinephrine system that regulates perception, arousal, and attention^25^. Such sympathovagal imbalance may result in reduced vigilance and attentional resources available for environmental contingencies, reflecting a tonic hypoarousal in ADHD^24^. Of note, one recent study indicated that acute MAE resulted in increased LF/HF ratio driven by parasympathetic withdrawal shortly after (i.e., 15–20 min) exercise cessation in children with ADHD. Authors of the study interpreted their findings as a favorable change of sympathovagal balance and prefrontal cortex activity induced by acute aerobic exercise^26^. Given the close relationship between prefrontal brain function and sympathovagal control^22^ accompanied by a sympathovagal imbalance in children with ADHD^24^, it is appealing to have a sustained measurement of HRV following acute exercise in this particular group to gain better insight of the mechanisms underlying inhibitory control.

Taken together, the objective of the current study was to investigate the sustained effects of acute MAE on inhibitory control via task performance, neuroelectric, and HRV measures in children with ADHD. The current study is novel in combining these measures to better understand the benefits of acute MAE on cognition and ANS modulations. It was hypothesized that acute MAE would result in better inhibitory control performance, accompanied by larger N2 and P3 amplitude reflecting the optimization of neuroelectric processes underlying improvements in cognition among a sample of children characterized by lower attentional inhibition capabilities. Further, it was hypothesized that acute MAE would result in increased post-exercise LF/HF ratio^26^ relative to baseline and when compared to a physically inactive control intervention, reflecting more favorable ANS modulations during post-exercise recovery. From a public health standpoint, the current investigation provides practical insight into the utility of acute bouts of MAE as a complementary behavioral treatment to manage ADHD symptoms. Moreover, a better understanding of whether acute MAE modulates neurocognitive correlates subserving core deficits of ADHD (e.g., deficit in inhibitory and attentional control, hypoarousal) may provide stronger clinical significance for the utility of acute bouts of exercise and may be informative in further designing effective non-pharmacological interventions.

## Results

Table 1 summarizes demographic characteristics of all participants. Preliminary analysis did not reveal any *Order* effects or interactions (*p’s ≥ *.07) with the exception of an *Intervention* × *Order* interaction for N2 amplitude (*F*(1, 22) = 16.52, *p* = .001, η^2^_p_ = .39) and N2 latency (*F*(1, 22) = 9.50, *p* = .005, η^2^_p_ = .30). Accordingly, *Order* was included as a covariate for all analyses on N2 measures. Table 2 summarizes behavioral and ERP outcomes across interventions, congruency trials, and time points.

**Table 1:** Participants’ descriptive characteristics.

Characteristics	M (SD)	Range
Gender (boys/girls)	23/1	
Age (years)	9.9 (1.3)	8–12
BMI (kg/m^2^)	18.0 (3.3)	13.7–26.7
IQ	105 (9.8)	92–128
SES (scale)	6.3 (0.8)	5–7
PDS (scale)	3.2 (0.5)	3–5
PACER (laps)	17.0 (8.4)	6–41
**Subtype of ADHD**		
Inattentive	5	
Impulsive	1	
Combined	18	
**Medication (n = 8)**		
Ritalin	6	
Concerta	1	
Atomoxetine	1	

*BMI* body mass index, *IQ* intelligence quotient, *SES* socioeconomic status, *PDS* Pubertal Developmental Scale, *PACER* Progressive Aerobic Cardiovascular Endurance Run Test.

**Table 2:** Summary of behavioral and ERP outcomes.

	30 min			60 min		
	Congruent	Incongruent	Interference score	Congruent	Incongruent	Interference score
**ACC (%)**						
Exercise	93.3 (4.9)	86.8 (8.6)	− 0.1 (0.1)	93.8 (4.5)	88.1 (6.7)	− 0.1 (0.0)
Video	87.5 (9.5)	80.8 (9.2)	− 0.1 (0.1)	86.6 (12.2)	80.0 (12.6)	− 0.1 (0.1)
**RT (ms)**						
Exercise	529.6 (80.6)	584.4 (88.2)	54.7 (24.8)	549.3 (82.7)	589.1 (90.8)	39.7 (29.2)
Video	526.5 (103.9)	585.1 (113.9)	58.6 (26.8)	544.3 (96.5)	595.0 (92.1)	50.8 (24.6)
**N2 amplitude (µV)**						
Exercise	− 11.6 (8.3)	− 11.7 (7.2)		− 11.5 (7.7)	− 10.8 (7.1)	
Video	− 9.9 (6.5)	− 9.3 (5.7)		− 9.1 (6.0)	− 9.7 (6.2)	
**N2 latency (ms)**						
Exercise	276.8 (27.9)	292.5 (38.4)		282.6 (30.7)	295.6 (37.9)	
Video	289.9 (29.4)	299.0 (31.9)		290.1 (35.0)	299.5 (45.0)	
**P3 amplitude (µV)**						
Exercise	10.8 (6.7)	11.0 (6.5)		6.7 (6.2)	9.3 (6.3)	
Video	10.1 (7.5)	12.6 (8.2)		8.7 (6.6)	8.8 (8.4)	
**P3 latency (ms)**						
Exercise	490.3 (87.4)	507.2 (98.8)		485.9 (98.6)	482.3 (89.6)	
Video	469.2 (107.0)	489.1 (84.9)		487.5 (102.9)	504.9 (104.7)	

*ACC* response accuracy, *RT* reaction times.

### In-exercise measures

The data indicated that mean heart-rate (HR) and ratings of perceived exertion (RPE) during exercise were 149.2 ± 4.9 bpm and 5.0 ± 2.6, respectively, indicating that children exercised at a moderate intensity.

### Behavior outcomes

Table 3 summarizes the statistics for the behavioral outcomes. The RM ANOVA on response accuracy showed a significant main effect of *Intervention*, *F*(1, 23) = 15.03, *p* = .001, η^2^_p_ = .40, with the exercise intervention (90.4 ± 4.7%) having higher response accuracy than the video intervention (83.7 ± 9.2%) regardless of time and congruency, as well as a significant main effect of *Congruency*, *F*(1, 23) = 67.48, *p* < .001, η^2^_p_ = .75, with congruent trials (90.3 ± 5.9%) having higher overall accuracy than incongruent trials (83.9 ± 6.7%). Supplemental analysis on accuracy interference did not reveal any significant main effect or interaction *F’s*(1, 23) = 0.16–0.28, *p’s ≥* .60*.*

**Table 3:** Summary of statistical analyses on behavioral, ERP, and HRV measures.

Variable	df	F	p	η^2^_p_
**Mean ACC**				
Session	1,23	15.03	.001*	.40
Time	1,23	0.00	.965	.00
Congruency	1,23	67.48	< .001*	.75
Session × time	1,23	0.53	.474	.02
Session × congruency	1,23	0.16	.695	.01
Time × congruency	1,23	0.28	.600	.01
Session × time × congruency	1,23	0.24	.630	.01
**ACC interference**				
Session	1,23	0.16	.695	.01
Time	1,23	0.28	.600	.01
Session × time	1,23	0.24	.630	.01
**Mean RT**				
Session	1,23	0.00	.965	.00
Time	1,23	2.76	.110	.11
Congruency	1,23	279.27	< .001*	.92
Session × time	1,23	0.01	.908	.00
Session × congruency	1,23	1.75	.198	.07
Time × congruency	1,23	5.45	.029*	.19
Session × time × congruency	1,23	0.54	.468	.02
**RT interference**				
Session	1,23	1.75	.198	.07
Time	1,23	5.45	.029*	.19
Session × time	1,23	0.54	.468	.02
**N2 amplitude**				
Session	1,22	7.55	.012*	.26
Time	1,22	0.22	.647	.01
Congruency	1,22	1.31	.264	.06
Session × time	1,22	0.01	.942	.00
Session × congruency	1,22	0.20	.662	.01
Time × congruency	1,22	0.01	.926	.00
Session × time × congruency	1,22	0.01	.931	.00
**N2 latency**				
Session	1,22	5.44	.029*	.20
Time	1,22	0.00	.976	.00
Congruency	1,22	1.29	.268	.06
Session × time	1,22	0.18	.673	.01
Session × congruency	1,22	2.02	.169	.08
Time × congruency	1,22	0.31	.584	.01
Session × time × congruency	1,22	0.46	.503	.02
**P3 amplitude**				
Session	1,23	0.23	.637	.01
Time	1,23	15.88	.001*	.41
Congruency	1,23	11.31	.003*	.33
Session × time	1,23	0.04	.849	.00
Session × congruency	1,23	0.01	.927	.00
Time × congruency	1,23	0.00	.995	.00
Session × time × congruency	1,23	14.60	.001*	.39
**P3 latency**				
Session	1,23	0.14	.708	.01
Time	1,23	0.03	.869	.00
Congruency	1,23	3.87	.061	.14
Session × time	1,23	1.85	.187	.07
Session × congruency	1,23	1.02	.324	.04
Time × congruency	1,23	0.98	.333	.04
Session × time × congruency	1,23	0.78	.386	.03
**HF-HRV**				
Session	1,20	0.45	.508	.02
Time	2,19	1.50	.249	.14
Session × time	2,19	4.65	.023*	.33
**LF-HRV**				
Session	1,20	1.93	.180	.09
Time	2,19	2.03	.159	.18
Session × time	2,19	4.58	.024*	.33
**LF/HF ratio**				
Session	1,20	0.00	.979	.00
Time	2,19	0.21	.811	.02
Session × time	2,19	0.09	.919	.01

*RT* reaction times, *HF-HRV* high-frequency heart-rate variability, *LF-HRV* low-frequency heart-rate variability.

*p < .05. ACC = response accuracy.

For mean RT, RM ANOVA showed a significant main effect of *Congruency*, *F*(1, 23) = 279.27, *p* < .001, η^2^_p_ = .93, with congruent trials (537.4 ± 84.3 ms) having shorter mean RT than incongruent trials (588.4 ± 90.2 ms). The main effect of *Congruency* was superseded by a *Congruency* × *Time* interaction, *F*(1, 23) = 5.45, *p* = .029, η^2^_p_ = .19. Decomposition of the interaction revealed that mean RT for congruent trials was shorter than incongruent trials at the post-30 time point (congruent: 528.1 ± 86.4 ms vs. incongruent: 584.7 ± 96.3 ms) and the post-60 time point (congruent: 546.8 ± 86.2 ms vs. incongruent: 592.1 ± 89.0 ms). Supplemental analysis on RT interference showed a significant main effect of *Time*, *F*(1, 23) = 5.45, *p* = .029, η^2^_p_ = .19, with the post-30 time point (56.7 ± 20.6 ms) having larger RT interference than the post-60 time point (45.2 ± 17.5 ms).

### ERP outcomes

**Figure 1:**
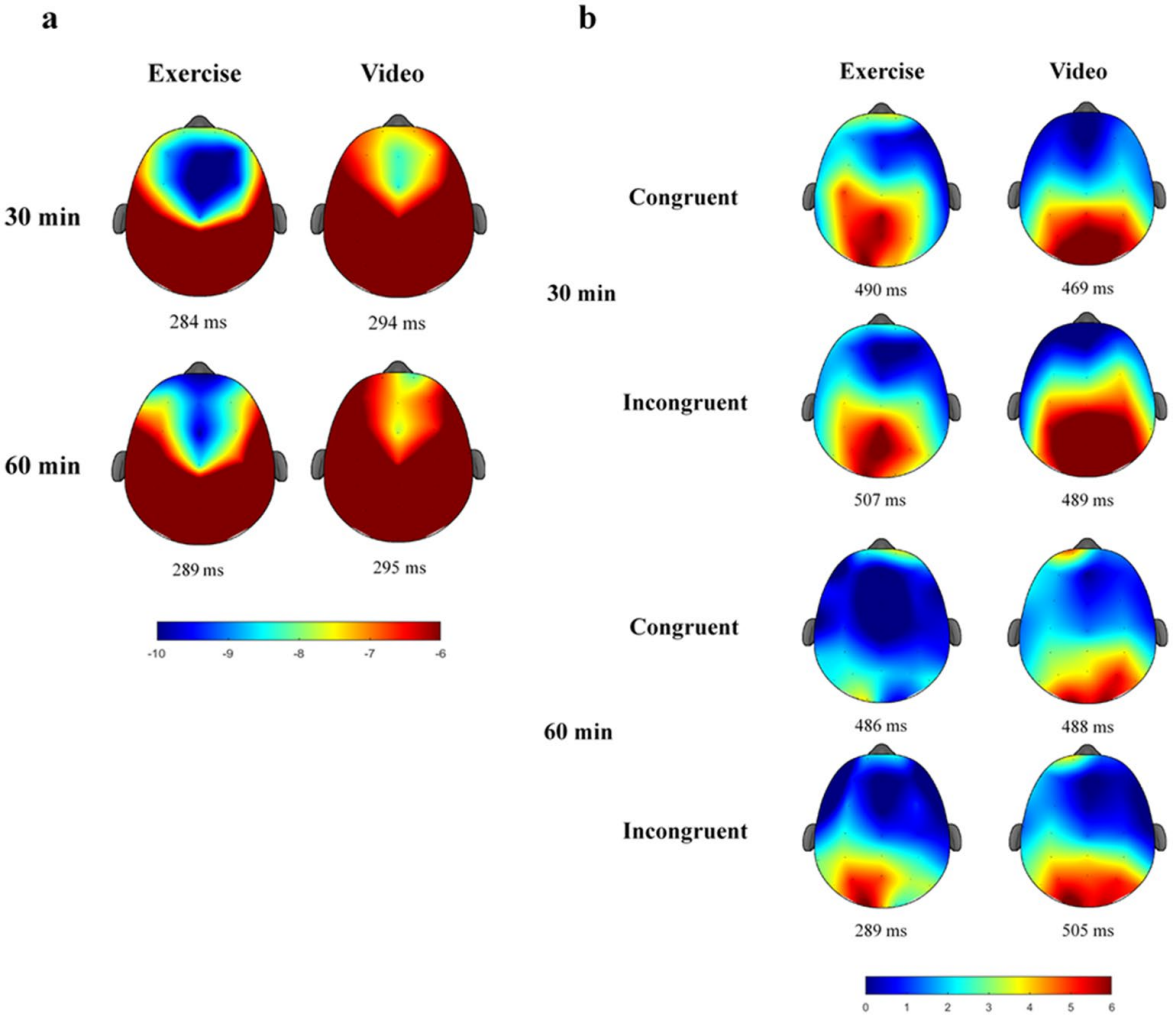
Topographical distribution (spectrum: blue to red) of N2 amplitude as a function of intervention and time (**a**) and P3 amplitudes as a function of intervention, time, and congruency (**b**). Figure was created by EEGLAB v2019.0 (https://sccn.ucsd.edu/eeglab/index.php).

**Figure 2:**
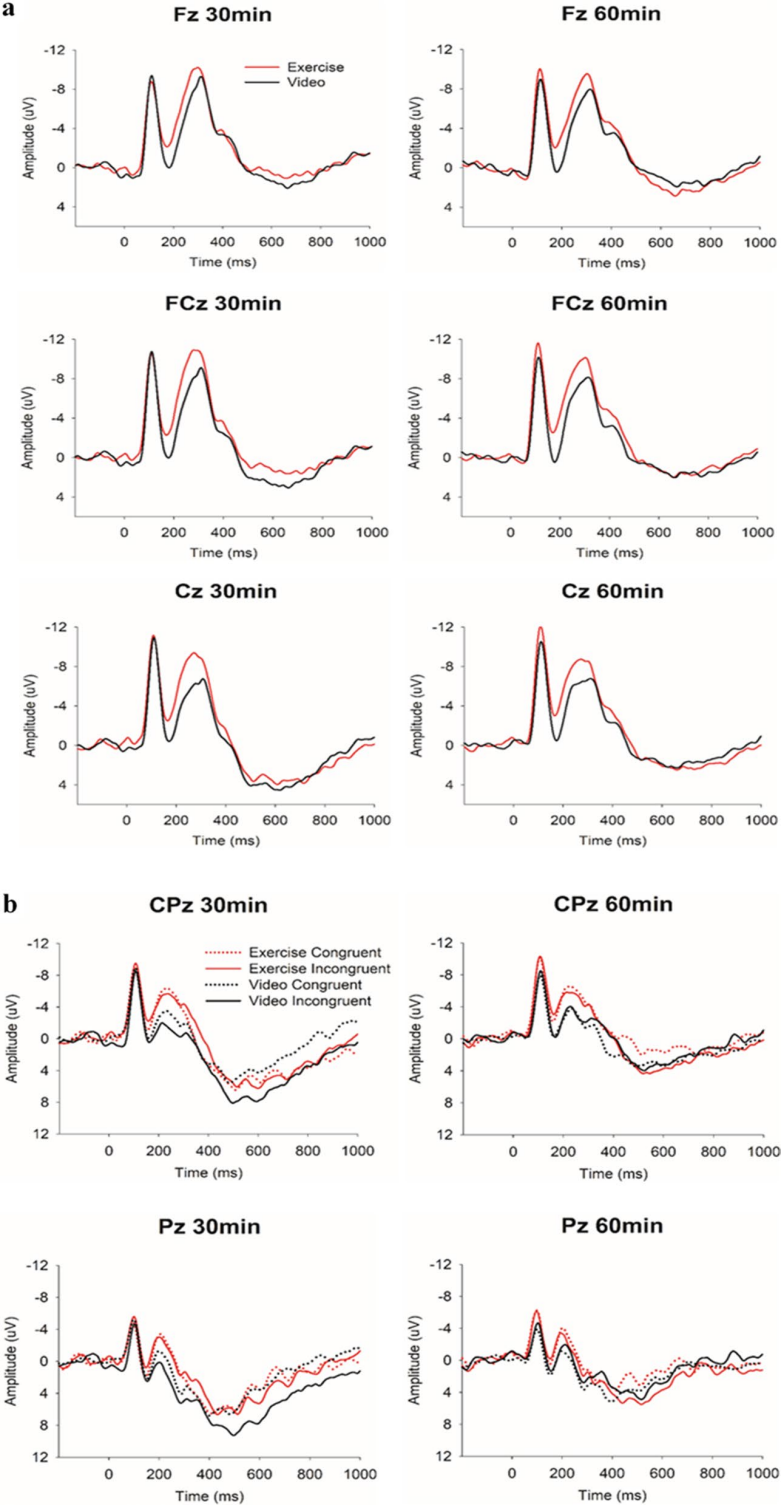
Grand-averaged waveforms of the N2 (**a**) and P3 component (**b**). Data of N2 were collapsed across congruency trials.

Table 3 summarizes statistics for N2 and P3 measures. The RM ANOVA on N2 amplitude showed a significant main effect of *Intervention*, *F*(1, 22) = 7.55, *p* = .012, η^2^_p_ = .26, with the exercise intervention (− 11.4 ± 7.2 µV) having larger N2 amplitude than video-watching intervention (− 9.5 ± 5.7 µV). N2 latency also showed a main effect of *intervention*, *F*(1, 22) = 5.44, *p* = .029, η^2^_p_ = .20, with the exercise intervention (286.9 ± 28.8 ms) having shorter N2 latency than video intervention (294.6 ± 31.6 ms). Figures 1a and 2a depict the topographic plots and grand-averaged waveforms of N2-ERP.

The RM ANOVA on P3 amplitude showed a significant main effect of *Time*, *F*(1, 23) = 15.88, *p* = .001, η^2^_p_ = .41, with the post-30 time point (11.1 ± 6.1 µV) having larger P3 amplitude than the post-60 time point (8.4 ± 5.8 µV), and a significant main effect of *Congruency*, *F*(1, 23) = 11.30, *p* = .003, η^2^_p_ = .33, with congruent trials (9.1 ± 5.3 µV) having smaller P3 amplitude than incongruent trials (10.5 ± 6.2 µV). There was an *Intervention* × *Time* × *Congruency* interaction, *F*(1, 23) = 14.60, *p* < .001, η^2^_p_ = .39. Decomposition of the three-way intervention examined *Intervention* × *Congruency* within each time point. The subsidiary ANOVAs yielded significant *Intervention* × *Congruency* interaction within the post-30 time point, *F*(1, 23) = 5.24, *p* = .032, η^2^_p_ = .19, and post-60 time point, *F*(1, 23) = 6.19, *p* = .021, η^2^_p_ = .21. However, follow-up analyses revealed no Intervention effect across congruency trials within the post-30 time point (p’s = .30–.65) or the post-60 time point (*p*’s = .19–.73)*;* only a Congruency effect was observed following exercise within post-60 time point (congruent trials: 6.7 ± 6.2 µV vs. incongruent trials: 9.3 ± 6.3 µV; *p* < .001) and following video-watching within the post-30 time point (congruent trials: 10.1 ± 7.5 µV vs. incongruent trials: 12.6 ± 8.2 µV; *p* = .004). On the other hand, RM ANOVA on P3 latency did not reveal any main effect or interaction, *F’s*(1, 23) = 0.03–1.85, *p’s ≥ *.187, except a marginal *Congruency* effect, *F*(1, 23) = 3.87, *p* = .061 (congruent trials: 483.2 ± 98.0 ms vs. incongruent trials: 495.9 ± 93.9 ms). Figures 1b and 2b illustrate the topographic plots and grand-averaged waveforms of P3-ERP.

### HRV outcomes

**Figure 3:**
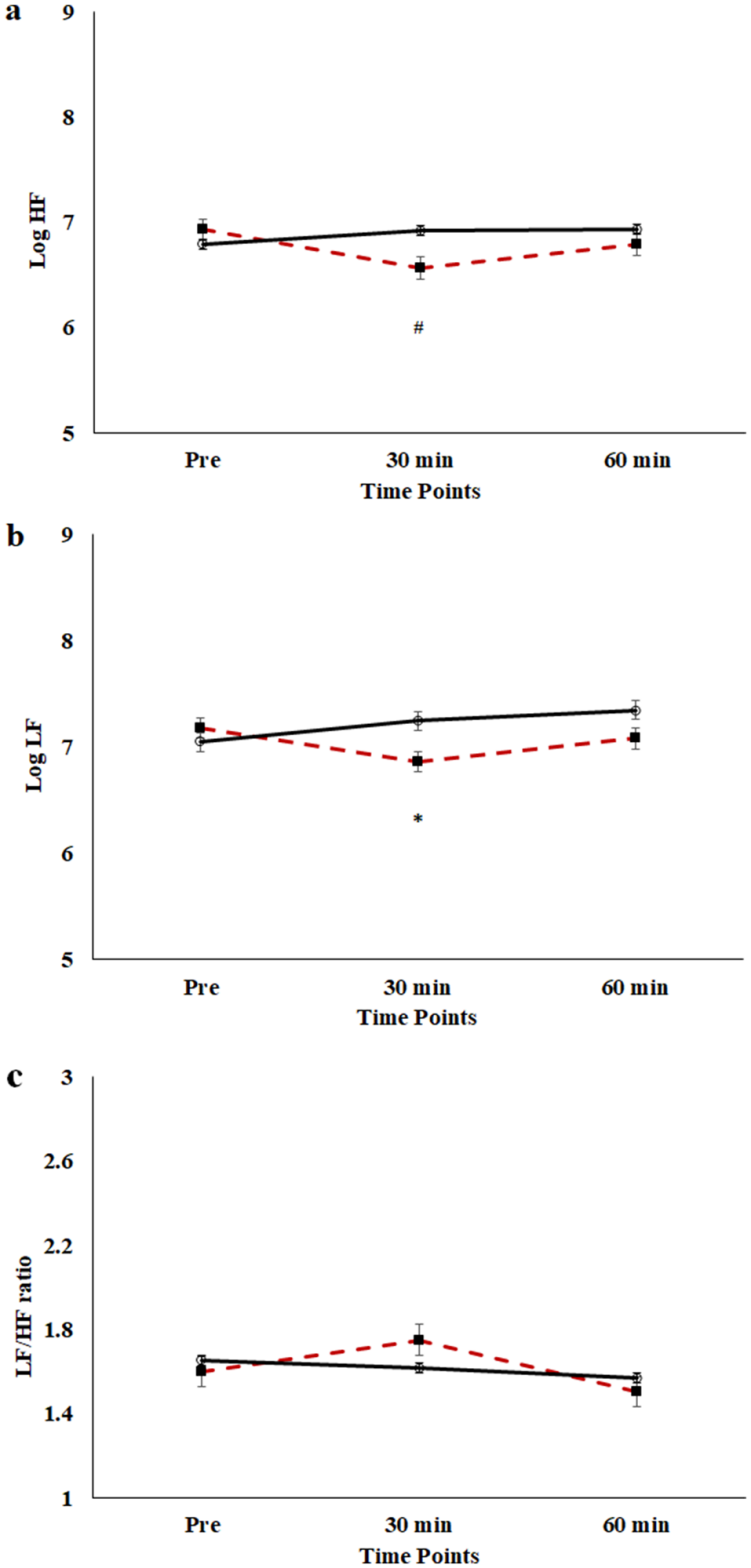
Changes in log-transformed HF-HRV (**a**), and LF-HRV (**b**) and LF/HF ratio (**c**) as a function of intervention and time. Data are presented as the mean ± SE. Red-line: exercise intervention. Black-line: video intervention. ^#^differ from baseline time point, *differ between intervention.

See Table 3 for the statistical summary of HF-HRV, LF-HRV, and LF/HF ratio. The RM ANOVA on HF-HRV revealed a significant *intervention* × *Time* interaction, *F*(2, 19) = 4.65, *p* = .023, η^2^_p_ = .33. Follow-up analyses indicated that HF-HRV at post-30 (6.6 ± 0.7) was smaller than baseline (6.9 ± 0.6) for the exercise intervention. There were no differences between baseline and post-60, post-30 and post-60, or difference between exercise and video intervention at post-30 and post-60 time point (*p’s ≥* .112). The RM ANOVA on LF-HRV showed a significant *intervention* × *Time* interaction, *F*(2, 19) = 4.58, *p* = .024, η^2^_p_ = .33. Follow-up analyses indicated smaller LF-HRV (6.8 ± 0.6) following exercise intervention relative to video intervention (7.3 ± 0.8) at post-30 time point. However, the RM ANOVA on LF/HF ratio did not reveal any significant main effect or interaction *F’s*(2, 19) = 0.01–0.21, *p’s ≥* .81*.* Figure 3 depicts fluctuations of HRV, including HF-HRV, LF-HRV, and LF/HF ratio as a function of intervention and time.

### Bivariate correlations

**Figure 4:**
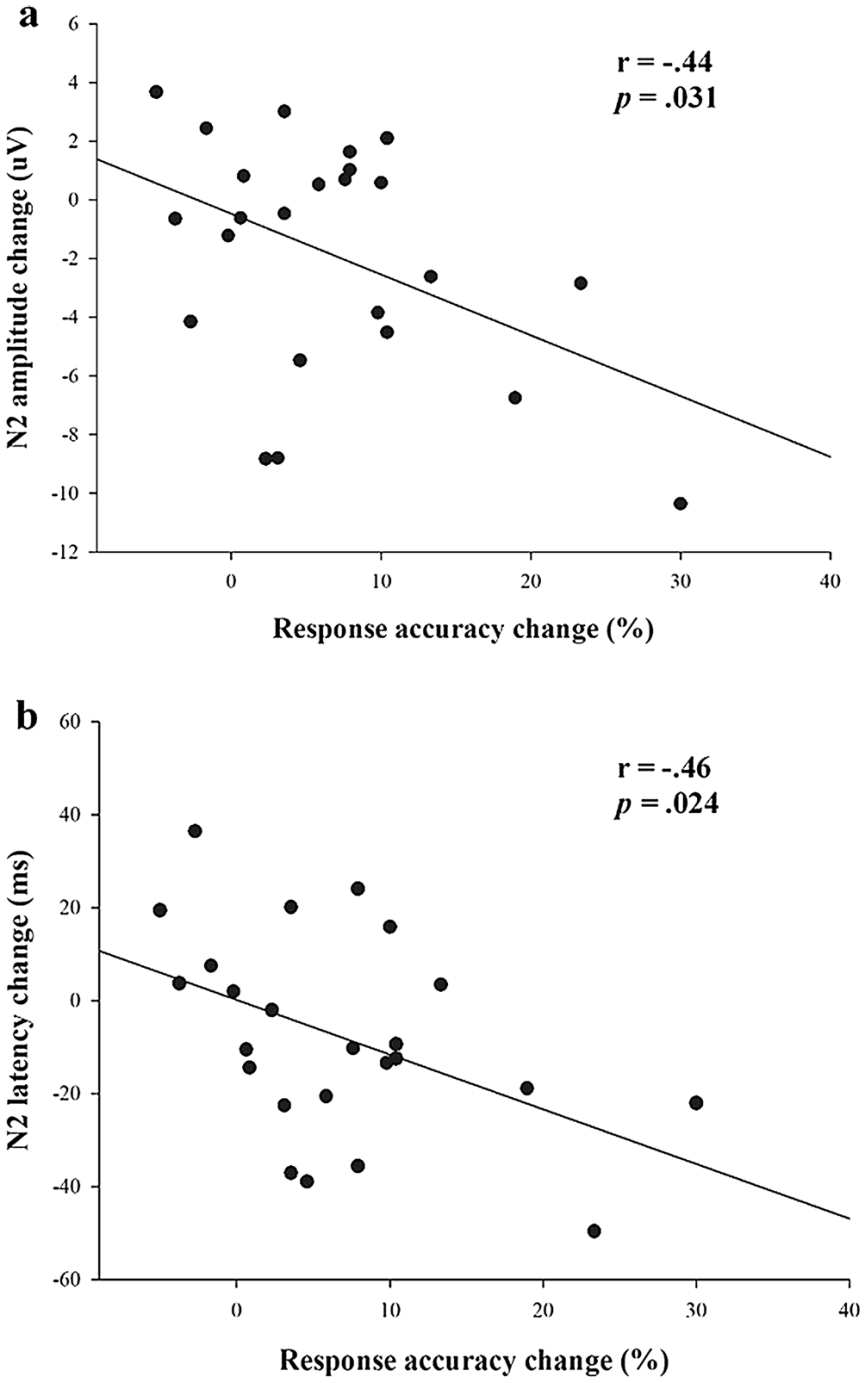
Scatter plots depicting the association between change scores in response accuracy and N2 amplitude (**a**), and the association between changes scores in response accuracy and N2 latency (**b**). Data were collapsed across congruency trials and time points.

Given the significant MAE effects on response accuracy and N2-ERP across time points and congruency trials, bivariate correlations were separately calculated between changes in response accuracy and changes in N2 amplitude as well as changes in response accuracy and changes in N2 latency by collapsing data across time points and congruency trials. Results revealed that changes in response accuracy were inversely correlated with changes in N2 amplitude (r = − .44, *p* = .031) and changes in N2 latency (r = − .46, *p* = .024), respectively, suggesting that greater improvements in overall accuracy were correlated with larger (more negative) increases N2 amplitude and greater decreases in N2 latency regardless time or congruency trials. Figure 4 presents scatter plots depicting the associations between changes in response accuracy and changes in N2-ERP.

## Discussion

The present findings revealed that acute bouts of MAE resulted in higher response accuracy of a modified flanker task across congruency and time. Acute MAE further resulted in larger N2 amplitude and shorter N2 latency irrespective of congruency and time, suggesting improved conflict detection. Moreover, bivariate correlations suggest that changes in N2 amplitude and latency were correlated with changes in response accuracy irrespective of time and congruency. However, in terms of the P3, our data indicated that acute MAE has no effect regardless of task difficulty or time. Regarding HRV outcomes, although lower HF-HRV and LF-HRV were observed following acute MAE for about 30 min, there was no change in LF/HF ratio, suggesting that acute MAE has no sustained effect on sympathovagal balance. Overall, our data imply that in children with ADHD, although acute MAE benefits behavioral performance and conflict detection during a flanker task for 60 min after following exercise cessation, ANS modulations might not be related to the associated behavioral and neuroelectric modulations induced by acute MAE.

Relative to the behavioral findings, the current study is novel in showing that acute MAE enhances flanker task performance, in terms of higher response accuracy, irrespective of cognitive loads for about 60 min in children with ADHD. Previously, research suggested a general benefit of acute bouts of MAE to inhibitory control performance in children with ADHD. That is, acute MAE enhanced inhibitory control, in terms of higher response accuracy^12^ or faster response speed^11,13,14^ across task components requiring variable cognitive demands. It is noteworthy that all prior studies in ADHD children examined modulations of inhibitory control either immediately after (i.e., within 5 minutes^13,14^) or after a short delay (i.e., < 15 min^11,12^) relative exercise cessation. The current findings, however, corroborate the observed general benefits of acute MAE to behavioral outcomes of inhibitory control, and extend the knowledgebase by providing evidence supporting the sustained effects of acute MAE on inhibitory control in children with ADHD. Such findings enhance the practical relevance of acute MAE as a complementary treatment to children with ADHD.

One novel finding of the current investigation is that acute MAE resulted in larger N2 amplitude and shorter N2 latency across congruency trials and time, suggesting that children with ADHD seemed to have more effective conflict detection following exercise for about 60 min. Despite no previous reports of N2 modulation following acute exercise in children with ADHD, our finding is consonant with prior research indicating larger N2 amplitude following acute MAE in individuals with deficit in inhibitory control (i.e., methamphetamine-dependent patients^18,19^). This enlarged N2 amplitude, coupled with shorter N2 latency and higher response accuracy, suggest that acute MAE may influence underlying mechanisms of inhibitory control via improved conflict detection during a flanker task in children with ADHD. This argument is supported by significant correlations between greater improvements in overall response accuracy and larger increases in overall amplitude of N2 or larger decreases in overall latency of N2 following MAE, implying that the N2-ERP could be a tangible neural marker when examining the prolonged effects of acute MAE on performance in task that modulates inhibitory control in children with ADHD. To the best of our knowledge, the current study is the first to demonstrate the prolonged effects of acute exercise on the N2 over time in children with ADHD. Such acute MAE-induced changes in N2 could be driven by the modulatory effects of acute exercise on the dorsolateral prefrontal cortex (DLPFC) and anterior cingulate cortex (ACC). Specifically, it has been well-documented that children with ADHD have hypoactivation in the DLPFC and ACC, two brain regions that account for conflict detection and behavioral adaptation^27^, during inhibitory control tasks^28^. Given that neuroimaging studies demonstrated that acute MAE improves cognitive control performance via increased activation in cortical networks involving the DLPFC^29^ and ACC^30^, it is possible that acute MAE engenders beneficial effects on conflict detection by affecting these two brain regions in children ADHD. Overall, our N2 findings provide stronger support for the relevance of acute MAE in managing inhibitory control deficits in children with ADHD.

With regard to the P3, although our data indicated an interaction involving exercise on P3 amplitude, there were no significant findings following post hoc analyses. This non-facilitative effect of acute MAE on P3 amplitude is in alignment with prior works showing larger N2 amplitude and unchanged P3 amplitude following acute MAE in young adults^31^ and methamphetamine-dependent patients^18,19^, implying that better conflict detection at the earlier stage of processing might be followed by less subsequent need for attentional control over an imperative stimulus. However, it is noteworthy that our findings contradict with prior studies on children with ADHD^11,12^. Such discrepancy may be accounted for, at least in part, by the specific demands of our modified flanker task, insufficient power to detect exercise effect, or the timing of the cognitive assessments following acute exercise. For example, the flanker task used herein included a fixation cue, while the version used by Pontifex et al. did not^12^. Thus, differences in task design may shape how acute MAE exerts influence over stimulus–response interactions, resulting in differential ERP findings. Stated differently, the pre-stimulus fixation and short ITI (i.e., 1000 ms) in our flanker task might induce early attention engagement or modified cognitive strategy (e.g., propensity to implement a proactive control strategy) in our participants^32,33^, which may, in turn, result in facilitated conflict detection, as signified by N2, and smaller attention engagement during response selection. This speculation could be supported by our relatively smaller overall P3 (9.8 µV) compared to prior study also on children with ADHD (12.7 μV^11^). Alternatively, the non-significant effect of acute exercise may simply reflect type two error given the relatively low statistical power resulting from a repeated measured design and relatively small sample size. Further, past studies assessed participants’ cognitive performance immediately following or after a short delay relative to exercise cessation, whereas we examined children’s cognitive performance at least 30 min after exercise. In the current study, it is plausible that the beneficial effects of acute MAE on inhibitory control may have begun to subside at the time of cognitive assessment.

In addition to behavioral and ERP measures, the current study leveraged the close relationship between prefrontal-mediated cognitions and sympathovagal control to better understand how acute aerobic exercise affects inhibitory control and ANS function over time in children with ADHD. Despite acute MAE altered HF-HRV and LF-HRV during the post-exercise recovery, there was no such modulation in LF/HF ratio indicative of sympathovagal balance across time or intervention, suggesting that acute bout of exercise might not induce changes in resting-state sympathovagal balance following exercise cessation. Previously, Ludyga et al. found that acute aerobic exercise resulted in increased LF/HF ratio during a cognitive flexibility task in children with ADHD^26^, implying a favorable change in ANS modulations and arousal. It is noteworthy that HRV measures were taken place within 20 min following exercise in Ludyga et al. whereas HRV data were collected at the 30- and 60-min time point following exercise in the current study^26^. During the post-exercise period, it is plausible that changes in sympathetic and/or parasympathetic tones sustained for a short period of time and returned to baseline levels after a delay. This assumption could be supported by another study from Ludyga and colleagues that indicated that LF/HF ratio during a flanker task increased immediately following acute exercise and returned to baseline between 30 and 60 min following exercise cessation in a group of adolescent children^34^. Collectively, the current HRV findings, together with the behavioral and neuroelectric findings, suggest that acute bout of aerobic exercise could facilitate flanker task performance and conflict detection for 60 min in children with ADHD; however, ANS modulations might not account for the associated behavioral and neuroelectric modulations induced by aerobic exercise. Given that we only collected data at the 30- and 60-min time points during post-exercise recovery, our data do not speak to the associations between acute MAE, resting HRV, and cognition immediately or shortly after exercise cessation.

The strength of the current study includes the novel approach to encompassing an inhibitory control task along with measurement of the N2- and P3-ERP and HRV to study the sustained effects of acute MAE on cognition and ANS modulations in children with ADHD. However, despite this strength there are several limitations to be acknowledged. First, it was unexpected that most of our participants were boys who came from families with relatively high SES. Whereas there is no evidence that gender or SES is a strong moderator to the effects of acute exercise on cognition, it should be noted that the current findings may not generalize to girls or children from lower SES. Second, there was an unbalanced distribution of ADHD subtypes in our sample, with approximately 70% of participants diagnosed with the *combined* subtype (n = 17) and the other 30% diagnosed with either the *inattentive* (n = 6) or *impulsive* (n = 1) subtype. We decided not to examine whether ADHD subtype differentiated the effects of acute MAE on cognition given insufficient statistical power to compare differential effects of acute MAE across all three subtypes. Thus, future study is recommended to account for these factors. Lastly, eight children underwent physician’s treatment and were taking medication upon participation. Although data analyses with medication included as a between-subjects factor revealed neither a main effect or interaction involving medication (*p*’s ≥ .15, data not shown), one may argue that the non-significant results reflect type two error as a consequence of a relatively small sample size. However, we believe that the potential confounding effects from medication may have been mitigated in the current study given that (a) there was no differences between children with and without medication in terms of all demographic variables (*p*’s ≥ .14) and (b) as noted earlier, participants were required to refrain from medications for at least 24 h prior to each intervention. Regardless, future research with larger sample size and takes the potential confounding effects of medications into account is necessary.

In summary, the current study indicated that acute bouts of MAE facilitate inhibitory control and underlying neuroelectric process in children with ADHD for 60 min following exercise cessation. However, a single bout of MAE has no influence to ANS modulations signified by HRV during the post-exercise recovery period. To the best of our knowledge, the current investigation is one of the first to study the sustained effects of acute exercise on behavioral and neuroelectric correlates of cognition in children with ADHD using a multi-disciplinary approach that encompasses behavioral, neuroelectric, and cardiac outcomes. From a practical standpoint, although pharmacologic treatments are effective, they have several notable drawbacks. Thus, the current investigation highlights the efficacy of acute, moderate-intensity aerobic exercise as a potential complimentary and non-pharmacologic treatment for neurocognitive deficit in children with ADHD to better manage their symptoms, facilitate brain health, and optimize inhibitory control.

## Methods

### Participants

Thirty children (29 boys, 1 girl) with ADHD between the ages of 8 to 12 years old were recruited from the greater Taipei area. All participants met the following inclusion criteria: (1) previous diagnosis of ADHD by their pediatrician according to the 5th edition of the Diagnostic and Statistical Manual of Mental Disorders (DSM-5; American Psychiatric Association, 2013) criteria; (2) no history of brain injury or neurological conditions such as epileptic seizures, serious head injuries, or periods of unconsciousness; (3) free of comorbid conditions such as Asperger syndrome, Tourette syndrome or conduct/oppositional defiant disorder; (4) free of intellectual disability; (5) capable of performing exercise based on a preliminary screening and free of any medical conditions (e.g., heart and cardiac conditions that limit exercise, dizziness in association with exercise, chronic medical conditions, bone fracture, asthma) listed on the Physical Activity Readiness Questionnaire (PAR-Q)^35^, and (6) normal or corrected-to-normal vision.

With regard to demographic and anthropometric data of participants, the Pubertal Developmental Scale (PDS) is a 12-point scale that was used to measure participants’ pubertal timing based on growth spurts, body hair development, breast growth, and menarche, with scores < 4 indicating pre-pubertal stage, scores between 4 and 5 indicating early pubertal stages, and scores ≥ 6 indicating later pubertal stages^36^. The Chinese version of PDS has been shown to be a reliable and valid instrument of pubertal stage assessment^37^. Socioeconomic status (SES) calculated by the Family Affluence Scale (FAS), which was composed of four items (e.g., how many car/computers does your family own, how many times did you travel away on holiday with your family in a year, do you have your own bedroom for yourself). We categorized participants into three family affluence groups by FAS scores: low (0–3), medium (4–5), high (6–7 points)^38^. Previous studies have indicated that the Chinese version of FAS has good internal reliability and external validity in Taiwan^39^. Participants administered the Test of Nonverbal Intelligence-Third Edition^40^ by a trained experimenter to gain an estimate of intelligence quotient (IQ). Height and weight were measured in the laboratory, and body mass index (BMI) calculated as weight (kg)/height (cm^2^) as a surrogate measure of body composition. Before starting, a written informed consent and a written informed assent approved by the Institutional Review Board at National Taiwan Normal University were completed by the parents and children, respectively.

### Measurement

#### Aerobic capacity assessment

Aerobic capacity assessment took place at the university’s indoor basketball court. Aerobic capacity was measured by the Progressive Aerobic Cardiovascular Endurance Run Test (PACER; Human Kinetics, Champaign, IL). PACER is a multistage progressive 20 m shuttle run test, which has established validity in assessing aerobic capacity in children. Correlations between the PACER and traditional maximal oxygen consumption measures are strong (*r* = .83)^41^. After details of the PACER test were explained to the children, they were instructed to run between two lines 20 m apart which included a cadence delivered by a CD emitting audible signals (a “beep” sound) at prescribed intervals. In the first minute, the initial running speed was set at 8.5 km/h, which was subsequently increased by 0.5 km/h each minute. When a child failed to keep up with the pace by being unable to reach the line at the time of the tone, the test was terminated at the second fault, and the number of laps completed was recorded. Aerobic capacity was determined by the number of laps completed at the time the test was terminated due to failure to cross the line prior to the signal or volitional exhaustion.

#### Modified flanker task

Participants completed a modified version of the Eriksen flanker task^42^ to assess inhibitory control by substituting goldfish rather than letters as stimuli^12^. In this task, participants were instructed to respond as accurately as possible to the direction of a centrally presented target goldfish amid either congruous (the target faced the same direction  ) or incongruous (the target faced the opposite direction  ) flanking goldfish stimuli. Each trial began with a central fixation cross for 500 ms, followed by a black background for 500 ms. Next, the stimuli, which were 3 × 3 cm tall yellow fish, were presented for 200 ms with an inter-stimulus interval (ITI) of 1200 ms. Participants were asked to press a button (‘F’ or ‘J’ on the keyboard) to indicate whether the target stimulus pointed to the left or right. Stimulus timing was delivered using NeuroScan Stim software Version 2.0 (Neuro, Inc., Charlotte, NC, USA). A block of 40 practice trials was delivered, which required participants to meet a criterion of 75% accuracy (i.e., a minimum of 30 correct responses) before starting the actual test. Afterwards, participants completed 3 blocks of 80 trials presented with equiprobable congruency and directionality. The participants were provided with a 1-min break between blocks. The total duration of the task was approximately 12 min. For behavioral outcomes, data on mean response accuracy and mean reaction time (mean RT) of correct responses were analyzed. Interference scores for response accuracy and mean RT were also calculated by subtracting the absolute value of congruent trials from incongruent trials.

#### Heart rate variability

HRV data were derived by spectral analysis of the inter-beat interval (R–R) series^43^. R-R interval data were recorded simultaneously using a V800 Polar HRM with a Polar H7 chest strap, at a sampling frequency of 1000 Hz (V800 Polar HRM; Polar Electro Oy, Kempele, Finland). Data were saved as R-R interval data files, with intervals timing provided in ms. Next, the data were preprocessed and analyzed using Kubios HRV (Biosignal Analysis and Medical Imaging Group, University of Eastern Finland, Joensuu, Finland) for frequency domain components^44^. In the software, the R-R interval data with a 4 Hz cubic spline interpolation were converted to equidistantly sampled series. Afterwards, a linear detrend correction based on smoothness priors to regularization (0.001 Hz cutoff) was applied to the R-R series to remove slow nonstationary trends from the signal^45^. A 5-min period of resting-state data were subjected to fast Fourier transform (FFT) using a Welch’s periodogram (300 s with 50% overlap)^44^. Based on HRV analysis guidelines^46^, a minimal period of 2 min is considered satisfactory to estimate frequency domain measures from short-term recordings. Power spectral density values over the respiratory low frequency band (LF: 0.04–0.15 Hz) and high frequency band (HF: 0.15–0.4 Hz) were used to calculate LF-HRV, HF-HRV, and LF/HF ratio^47,48^. This measure of autonomic function has demonstrated good long-term temporal consistency^48^ and reliability^47^.

#### Electroencephalographic recordings

Electroencephalographic (EEG) activity was measured during the flanker task with 32 electrode sites using an elastic electrode cap (Quik-Cap, Compumedics Neuroscan, Inc., Charlotte, NC, USA) according to a modified International 10–20 System. Ongoing EEG activity was referenced to the average of the mastoids (M1, M2), with AFz serving as the ground electrode. Electrooculographic (EOG) activity was recorded using four electrodes placed at the outer canthus of each eye, and above and below the left orbit. All electrodes were maintained at impedances < 10 kΩ before data recording^49^. Continuous data acquisition was performed with a sampling rate of 1000 Hz, a DC- to 200-Hz filter, and a 60-Hz notch filter using a Neuroscan SynAmps2 amplifier^50^. Matlab (R2019a, Mathworks Inc.), EEGLAB toolbox (version 2019.0^51^), and ERPLAB toolbox (version 7.0.0^52^) were used for offline data processing. Continuous data were reduced by Independent Component Analysis and an automated eyeblink component removal procedure^53^. Next, the data were created from − 200 to 1000 ms relative to stimulus onset, baseline-corrected using the mean amplitude of the 200-ms window before stimulus, and filtered using a zero phase-shift low-pass filter (IIR Butterworth filter) at 30 Hz (24 dB/oct)^49^. Trial epochs were rejected if a response error occurred, an identified artifact exceeded ± 100 μV, or the overall variance of the epoch exceeded ± 3 standard deviations of the mean of local (by electrode) and global (all electrodes) accepted epochs. The mean number of trials included for analysis across intervention, task conditions, and time points were as follows: exercise-congruent-post 30: 95.2 ± 20.9; exercise-incongruent-post 30: 88.6 ± 18.6; exercise-congruent-post 60: 98.6 ± 19.3; exercise-incongruent-post 60: 91.8 ± 17; video-congruent-post 30: 92.7 ± 20.4; video-incongruent-post 30: 84.3 ± 20.5; video-congruent-post 60: 88.6 ± 26.3; video-incongruent-post 60: 81.8 ± 5). The latency and amplitude of N2 and P3 were quantified using peak latency and mean amplitude within a ± 25 ms interval surrounding the largest ongoing positive and negative peak within a fixed 200–400 ms and 300–700 ms post-stimulus latency window, respectively. Given that our preliminary analysis revealed larger (more negative) N2 amplitude at Fz, FCz, and Cz, which did not differ across sites (*p*’s ≥ .40), compared to CPz and Pz (*p*’s ≤ .03), as well as a topographical distribution centered at the frontocentral regions (Fig. 1a), the average of N2 indices across Fz, FCz, and Cz was used^49^. On the other hand, given there was larger P3 amplitude at CPz and Pz compared to Fz, FCz, and Cz (*p*’s ≤ .01) as well as a topographical distribution centered at the centroparietal regions (Fig. 1b), the average of P3 indices across CPz and Pz was used^54^. Please see Fig. 2 for grand-averaged waveforms for N2 and P3, respectively.

### Procedure

Participants visited the laboratory on two separate testing intervention (7 days apart). In order to minimize day-to-day variations in physiological and cognitive performance, participants were tested on the same day and time of the week, instructed to refrain from food and drink consumption except water for 1.5 h, and be free of medication and behavioral treatments for at least 24 h prior to each intervention^55^. Before the testing intervention, the experimental procedure was explained to participants and their legal guardians by experimenters. Then the legal guardians were asked to complete a health history, demographics questionnaire, SES, PDS, and an informed consent form. Once the demographic measures were completed, participants were instructed to sit on a chair in an electrically shield and sound-attenuated testing room where they were fitted with an electrode cap for the EEG recordings. Afterwards, participants were asked to rest for 5 min to collect baseline resting-state HR and HRV. Following the baseline resting-state HRV test, participants underwent either a 30-min treadmill walking/running or 30-min video-watching intervention. In the exercise intervention, they warmed up for 5 min on a motor-driven treadmill, then performed a 20-min bout of moderate-intensity aerobic exercise (defined as 60–70% of HR_reserve_). Target HR during exercise for each participant was pre-determined using the formula: ((HR_max_ − baseline HR) × 60–70%) + baseline HR, HR_max_ calculated using the formula 206.9 − (0.67 × Age)^56^, followed by a 5-min cool down. This protocol was selected by referring to previous studies demonstrating cognitive benefits of acute exercise in children with ADHD^11–13,57^, and was consistent with the exercise guidelines established by ACSM^56^. To ensure that children exercised at the pre-determined intensity, data on HR were measured every 2 min during exercise using a Polar watch. In addition, the OMNI ratings of perceived exertion (RPE), which ranges from 1 to 10, was also measured every 2 min to provide a subjective rating of individuals’ perceptions of their physiological efforts during exercise^58^. To examine the sustained effect of aerobic exercise on HRV and flanker task performance, participants then underwent a 5-min resting-state HRV measurement and the flanker task in fixed order, with both measurements administered at 30 min (post-30 time point) and 60 min (post-60 time point) following intervention. For the video-watching condition, participants were instructed to sit quietly and watch a video (emotionally neutral, relating to landscapes from aerial camera) in the same electrically shielded and sound-attenuated chamber for 30 min during the video-watching intervention. The decision of using a video-watching control intervention was based on relevant studies in the area^59,60^. After the video-watching intervention, children underwent the same procedure as for the exercise condition. Following completion of the flanker task at the post-60 time point in the video-watching intervention, participants then completed the Test of Nonverbal Intelligence and PACER test. Participants were given $20 compensation after they completed the two testing interventions. The study procedures and protocols reported herein were carried out in accordance with the declaration of Helsinki and arppoved by the Center for Research Ethics at National Taiwan Normal Univeristy (approval number: 201704HM003).

### Statistical analysis

Of the original 30 children, data on 6 children were discarded either due to an inability to complete the entire experimental protocol (n = 3), poor quality in EEG data (n = 1), or response accuracy below chance (i.e., less than 50% of response accuracy) in the inhibitory control task (n = 2). Moreover, HRV data from 4 participants were further discarded due to poor quality. With a remaining sample size of 24 for behavioral/ERP analyses and 20 for HRV analyses, the current investigation had sufficient sensitivity to detect repeated measures effects exceeding *f* = 0.20 for behavioral/ERP analyses and 0.24 for HRV analyses (assuming correlation between repeated measures ≥ 0.5), respectively, as computed using G^*^Power 3.1.9^61^. Of the remaining 24 children, 8 were taking stimulant medications (6 with Ritalin, 1 with Concerta, 1 with Atomoxetine). Children who took stimulant medications did not differ from those who did not in terms of age, BMI, IQ, SES, PDS, HR baseline, and PACER (*p*’s ≥ .14), and they were instructed to restrain from medications for at least 24 h prior to the experiment^55^. Therefore, we did not separate our participants based on medication. The remaining participants were of 23 boys and one girl. We did not exclude the one girl because sensitivity analysis comparing results between data with and without the only girl indicated that inclusion/exclusion of the girl did not change the results. Thus, we kept the one girl in all statistical analyses.

All statistical analyses were performed using SPSS 23.0 (IBM Corporation, Armonk NY, USA), with an alpha of .05 set as the significance criteria. Gaussian distribution of the data was verified with the Shapiro–Wilk Test. Preliminary analyses were performed to test whether the observed experimental effects were due to intervention order. As such, an additional between-subjects factor with two levels (*Order*: video-exercise, exercise-video) were included into the analyses described below. To examine the effects of acute aerobic exercise on behavioral, N2-ERP, and P3-ERP measures, several 2 (*Intervention*: exercise, video) × 2 (*Time*: post-30, post-60) × 2 (*Congruency*: congruent, incongruent) repeated-measure analyses of variance (RM ANOVAs) were performed on response accuracy, mean RT, and N2 and P3 measures. In addition, supplementary analyses on interference scores of response accuracy and mean RT were performed with several 2 (*Intervention*: exercise, video) × 2 (*Time*: post-30, post-60) RM ANOVAs once a significant *Congruency* effect was detected. For HRV outcomes, a 2 (*Intervention*: exercise, video) × 3 (*Time*: baseline, post-30, post-60) RM ANOVA was used. Greenhouse–Geisser correction was utilized if the assumption of sphericity was violated. Post hoc comparisons were corrected with *Bonferroni*-corrected *t*-tests. Partial eta square (η^2^_p_) effect sizes were reported in addition to significance testing, with η^2^_p_ of 0.01, 0.06, 0.14 indicating small, medium, and large effect sizes, respectively^62^.

To further investigate whether changes in ERP or HRV measures related to changes in behavioral outcomes, Pearson product-moment correlations were performed wherever significant MAE effects were detected. Data were obtained by subtracting data following video from MAE (exercise–video) for behavioral and ERP outcomes. Data of HRV outcomes were obtained by subtracting the relative change from baseline (post-30–baseline, post-60–baseline) in the video condition from relative change in MAE condition.
